# Data Processing of 2060-T8 Alloy Fatigue Test Results Using Statistical Methods to Improve Reliability and Accuracy

**DOI:** 10.3390/ma18081711

**Published:** 2025-04-09

**Authors:** Yuanbo Lv, Xianmin Chen, Youyou Fan, Yuxiang Tian, Feng Zhang

**Affiliations:** 1 National Key of Laboratory of Strength and Structural Integrity, AVIC Aircraft Strength Research Institute, Xi’an 710065, China; lvyuanbo040379@163.com (Y.L.);; 2 The 3rd Department, AVIC Aircraft Strength Research Institute, Xi’an 710065, China; 3 School of Mechanics, Civil Engineering and Architecture, Northwestern Polytechnical University, Xi’an 710129, China

**Keywords:** fatigue test, fatigue reliability, shot peening, statistical methods, aluminum–lithium alloy

## Abstract

Fatigue life testing is a crucial method for evaluating the fatigue performance of a material or part. However, the reliability and accuracy of the analysis methods applied to the data generated by such tests remain insufficient owing to their considerable scatter. This study accordingly conducted fatigue testing on fourth-generation 2060-T8 aluminum–lithium alloy specimens before and after low- and high-intensity cast steel shot peening. The resulting fatigue test data were evaluated for normality, processed using outlier identification, and subjected to hypothesis testing and comparative analysis. Finally, the fatigue reliability of each specimen group was calculated based on the significant differences between their observed fatigue lives. This study pioneers the systematic integration of normality testing, outlier identification, and hypothesis testing, constructing a multi-tiered analytical framework specifically tailored for shot peening effect evaluation. This approach fundamentally overcomes the constraints of traditional methodologies characterized by their sole reliance on mean fatigue life analysis. The determined effects of shot peening on the specimen fatigue life and data dispersion indicated that analysis using statistical methods can effectively improve the reliability of fatigue test results and support the selection of materials for use in engineering structures.

## 1. Introduction

The 2060-T8 alloy is a fourth-generation aluminum–lithium alloy that exhibits significant performance advantages over traditional aluminum alloys: its higher modulus of elasticity maintains better stability under the same external force [1], its significantly higher strength and stiffness facilitate structural weight reduction, and its excellent corrosion resistance extends its service life. Therefore, 2060-T8 is considered one of the most valuable lightweight, high-strength structural materials used in the aviation industry, representing a promising alternative to traditional aluminum alloys [2].

Shot peening is a common surface-strengthening treatment that has been widely applied to enhance the strength, fatigue resistance, and corrosion resistance of metal components. It comprises the high-speed projection of shot particles onto the surface of a workpiece to induce residual compressive stress in the material, thereby effectively increasing its fatigue strength [3]. A wide variety of shot peening processes using different shot materials, such as cast steel, glass, and ceramics, have been developed according to the intended working conditions and material properties. This study subjected 2060-T8 alloy workpieces to cast steel shot peening, which is particularly well-suited for improving the surface properties of such high-strength, wear-resistant materials [4].

Fatigue life testing is indispensable for evaluating the fatigue properties of materials and components and has long been a subject of significant research attention [5,6,7]. However, the fatigue lives of specimens subjected to the same treatment techniques and tested using the same test method on the same testing machine often exhibit considerable scatter [8]. As a result, researchers tend to pay more attention to the average fatigue life or limit values obtained from extensive testing of materials and components while neglecting to account for the impacts of random factors on specific fatigue test results and their influence on the reported average. Indeed, simply referring to mean or limit values fails to reflect the actual distribution of potential fatigue lives; a reliability analysis might provide a more complete understanding of fatigue life variability. As few reliability analyses have been conducted on fatigue test results, an in-depth statistical analysis of fatigue life data is required to better describe the true values of the test population, eliminate the interference of random factors, and improve the reliability of fatigue test results.

In this research, fatigue testing was carried out on 2060-T8 alloy specimens that were untreated, treated with low-intensity shot peening, and treated with high-intensity shot peening. The fatigue life data from each group were analyzed statistically and compared to explore how the shot peening treatment affected the fatigue performance of the alloy [9]. Normality tests were conducted to confirm that the data distribution characteristics met the requirements for statistical analysis, abnormal data were identified and removed to enhance reliability, hypothesis testing and comparative analyses of the data were performed to clarify the influence of shot peening on fatigue life, and the reliability life for each specimen group was calculated to provide a scientific basis for the engineering application of 2060-T8. The comparative analysis of fatigue performance conducted in this study revealed the impact of shot peening on the fatigue performance of the 2060-T8 alloy, providing effective and reliable support for the selection of structural materials and informing the optimization of shot peening process parameters. Thus, this paper represents a valuable reference for lightweight design in fields such as the aerospace industry [10].

## 2. Fatigue Testing

### 2.1. Specimen Design and Testing Method

**Figure 1:**
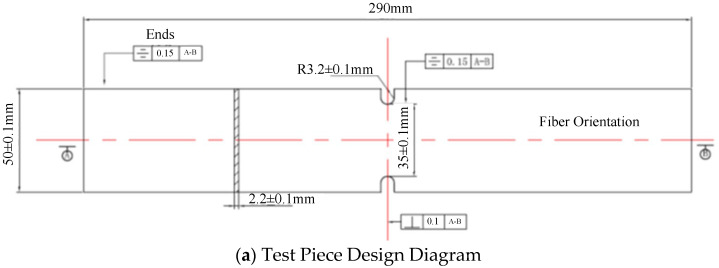

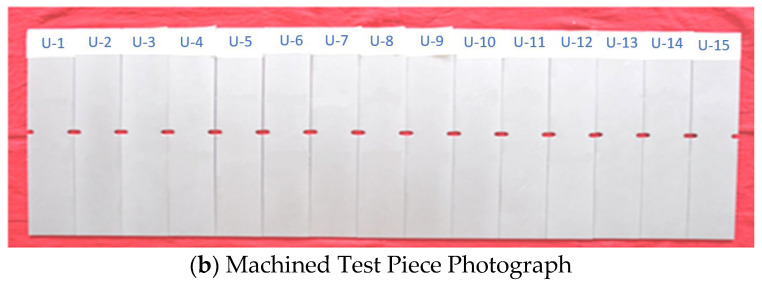
Schematic diagram of the test specimens.

Three groups of 2060-T8 alloy test specimens were subjected to fatigue testing: unpeened specimens, specimens treated with low-intensity shot peening, and specimens treated with high-intensity shot peening. Shot peening was conducted by applying a cast steel S230 shot to one plate of each type. Each plate was cut into 15 test specimens, as shown in Figure 1, totaling 45 specimens. The specific parameters of the test specimens and the corresponding shot-peening processes are listed in Table 1.

**Figure 2:**
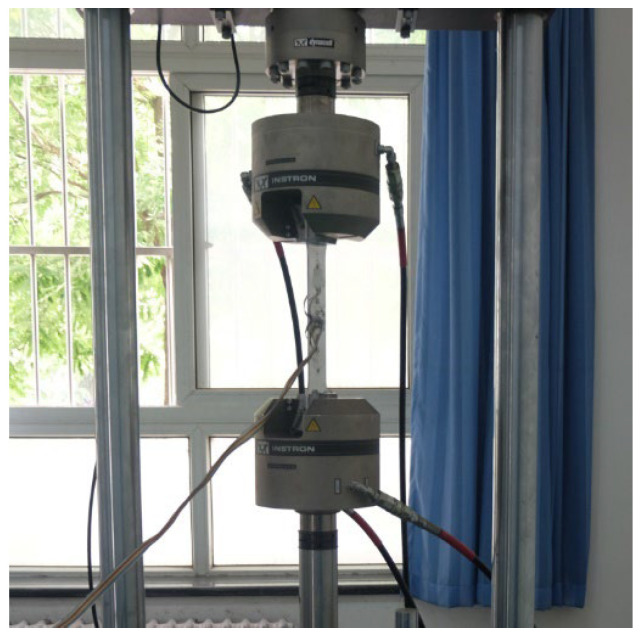
Specimen loading setup.

An INSTRON fatigue testing machine (Norwood, MA, USA) loaded each specimen using the setup shown in Figure 2 at room temperature according to the GB/T 3075–2008 standard [11]. A maximum load of 11 kN was applied to achieve a fatigue stress ratio of 0.1 at a loading frequency of 12 Hz. Strain gauges were pasted on the opposite faces of the test specimen, and the strain data were collected by an ST-16 data acquisition instrument. The coaxiality of the testing machine and test specimen was confirmed by comparing the strain data obtained from opposing gauges.

### 2.2. Test Results

**Figure 3:**
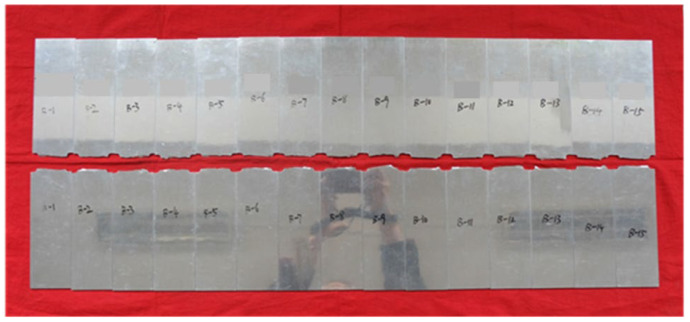
Typical specimen failure modes.

The number of fatigue cycles applied at the failure of each specimen, defined as complete tensile rupture according to GB/T 3075–2008, was recorded, and the results are listed in Table 2. The typical failure mode is illustrated in Figure 3.

## 3. Analysis and Processing of Fatigue Test Data

In-depth analyses of the fatigue test data were conducted to investigate the statistical laws describing the fatigue lives of the three specimen groups, assuming that the material properties of the same group of specimens were isotropic.

### 3.1. Distribution Testing

**Figure 4:**
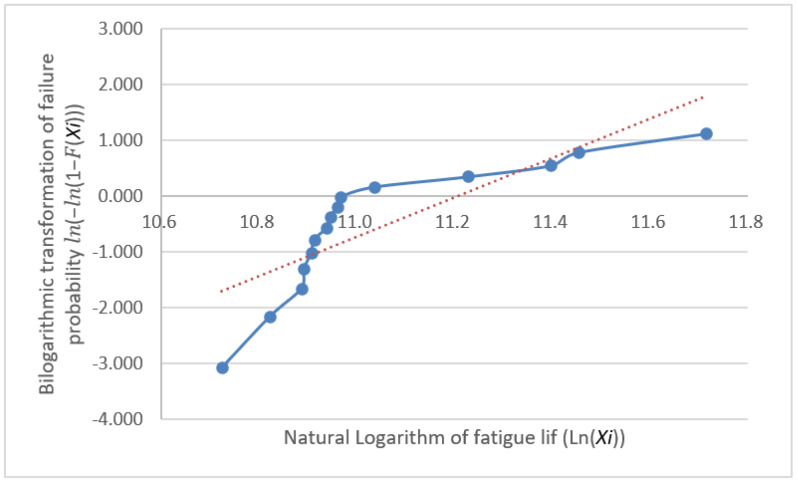
Group A natural logarithm fatigue life probability plots.

**Figure 5:**
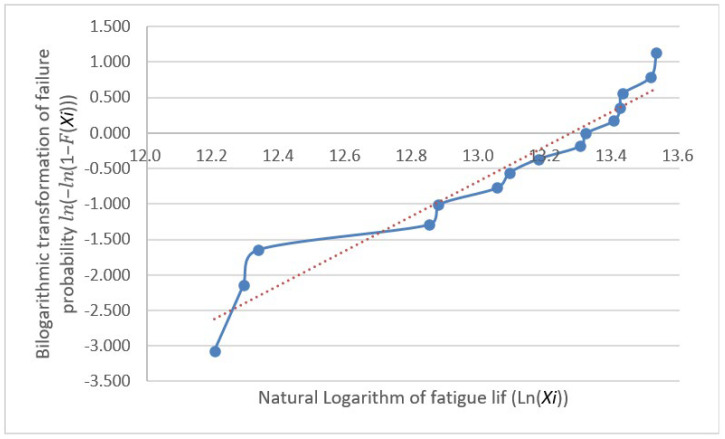
Group B natural logarithm fatigue life probability plots.

**Figure 6:**
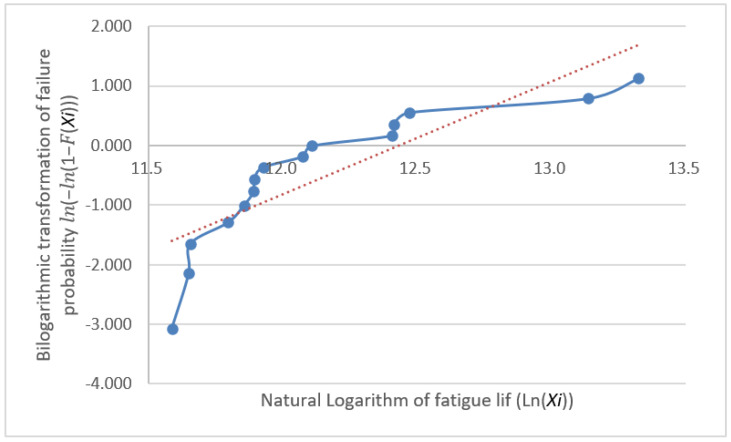
Group C natural logarithm fatigue life probability plots.

The fatigue life distribution for a group of specimens has been the focus of engineering research and is generally held to follow either a log-normal or Weibull distribution [12]. These distribution models are highly applicable to fatigue life data, as they effectively reflect the randomness and dispersion of data [13]. However, many classical statistical analysis techniques, such as hypothesis testing, analysis of variance, and regression analysis, require the analyzed data to follow a normal distribution [14] because it possesses favorable mathematical properties that simplify the calculation process and provide more reliable results for statistical inference. Thus, normality testing is not only a fundamental step in statistical analysis but also a crucial factor in ensuring the reliability and validity of the obtained results. Furthermore, it provides a rational basis for the selection of statistical models for fatigue life data analysis, enabling a more accurate assessment of fatigue performance and offering scientific support for engineering design and reliability evaluations. Subsequently, we conduct distributional verification procedures on the fatigue test dataset through separate Weibull distribution analysis and normality assessment. The analytical workflow initiates with a Weibull distribution compliance verification, employing probability plot-based graphical verification through the systematic implementation of Benard’s median rank method and linearized coordinate transformation, thereby enabling visual diagnostics of distributional adherence. First, the obtained fatigue life values were arranged in ascending order, that is $X_{1}\leq X_{2}\leq \cdots \leq X_{n}$; next, the cumulative distribution function $F(X_{i})$ for each specimen was calculated, where $F\left(X_{i}\right)=\frac{i-0.3}{n+0.4}(i=1,2,\ldots ,n)$; then, the bilinear coordinates $\left(ln\left(X_{i}\right),ln(-ln(1-F(X_{i})))\right)$) were plotted with a trend line. If these points are scattered along or near a straight line, the sample can be considered to follow a Weibull distribution. The plotted coordinates $\left(ln\left(X_{i}\right),ln(-ln(1-F(X_{i})))\right)$ for the data in Table 2 are shown with their fitting lines in Figure 4, Figure 5 and Figure 6.

The graphical validation through Weibull probability plot analysis reveals substantial deviation between the double-logarithmic coordinate points and the theoretical linear fit. This departure conclusively demonstrates non-conformance of the experimental fatigue dataset to the Weibull distribution paradigm.

Following the Weibull distribution conformity assessment, the analytical workflow proceeds to a normality verification phase. The probability paper plotting method was applied to conduct normality testing on the fatigue life data collected in this study as follows: first, the obtained fatigue life values were arranged in ascending order, that is $X_{1}\leq X_{2}\leq \cdots \leq X_{n}$; next, the cumulative distribution function $F(X_{i})$ for each specimen was calculated, where $F\left(X_{i}\right)=\frac{i-0.3}{n+0.4}(i=1,2,\ldots ,n)$; finally, the points $\left(X_{i},P\left(X_{i}\right)\right)$ were plotted on normal probability paper. If these points are scattered along or near a straight line, the sample can be considered to follow a normal distribution [15].

**Figure 7:**
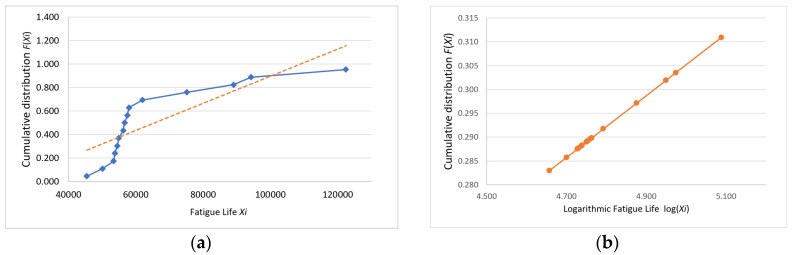
Group A (**a**) fatigue life and (**b**) logarithmic fatigue life probability plots.

**Figure 8:**
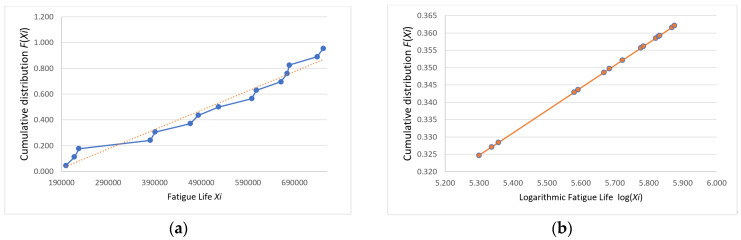
Group B (**a**) fatigue life and (**b**) logarithmic fatigue life probability plots.

**Figure 9:**
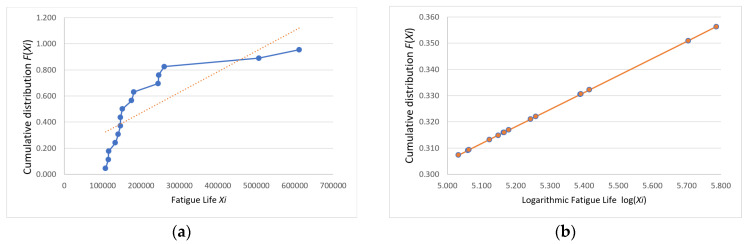
Group C (**a**) fatigue life and (**b**) logarithmic fatigue life probability plots.

The plotted coordinates $(X_{i},F\left(X_{i}\right)$) and $\left({log(X}_{i}\right),F\left(X_{i}\right))$ for the data in Table 2 are shown with their fitting lines in Figure 7, Figure 8 and Figure 9.

The fatigue life probability plots indicate that the logarithmic fatigue life values were closely distributed along a straight line, whereas the direct fatigue life values were not. Thus, the fatigue life data were determined to conform to a log-normal distribution, which provides a statistically reliable basis for analysis.

Meanwhile, non-parametric tests could provide robustness checks, especially for small sample sizes. Consequently, the study methodologically grounds its analytical framework by implementing the Kolmogorov–Smirnov (K-S) non-parametric goodness-of-fit test for systematic verification of distributional assumptions. Under the hypothesis that three datasets follow log-normal distributions, the K-S test was systematically implemented. Calculated K-S statistics for all datasets were found to be less than their respective critical values Dcrit=0.338 (α = 0.05), thereby statistically confirming adherence to log-normality. This validation satisfies the prerequisite for subsequent parametric analyses requiring log-normal distributional assumptions.

### 3.2. Processing Abnormal Fatigue Life Data

Fatigue tests often yield fatigue life values that are significantly lower or higher than the average value. These data are considered abnormal and can be produced by factors such as measurement errors, slight changes in the experimental conditions, and material inhomogeneities. Considerable deviations in the results of data analysis may occur if it is performed directly on a dataset containing abnormal values; these deviations can affect the accuracy of the fatigue life assessment. Therefore, all abnormal data must be removed.

Identification of abnormal data cannot rely solely on experience, but various statistical processing methods have been developed for this purpose; the common Chauvenet’s criterion was employed to do so in this study [16]. The specific procedure for data processing using Chauvenet’s criterion is as follows:

(1) Calculate the mean and standard deviation of the sample. These statistical measures serve as the basis for subsequent outlier determination.
(2) Calculate the deviation between each observed value and the mean, then divide this deviation by the standard deviation to obtain the “deviation ratio”, which reflects the degree of deviation between each data point and the overall sample.
(3) Using the standardized table for a log-normal distribution, find Chauvenet’s criterion value for which the probability of an observed value is less than 1/*n* (where *n* is the sample size).
(4) Compare the deviation ratio for each observed value with Chauvenet’s criterion value. If the former is greater than the latter, the observed value can be regarded as an outlier.

When there were more than two outliers in the data, the value with the larger error was removed, and then the mean and standard deviation of the remaining data were recalculated. The process was repeated until no outliers remained in the data. The specimen group number was updated with each such iteration, i.e., Group A was changed to A1. As shown in Table 3, i is the test piece number in each group, the fatigue life data dispersion (standard deviation) for the unpeened specimen group was clearly smaller than that for the shot-peened specimen groups, and that for the low-intensity shot-peened group was greater than that for the high-intensity shot-peened group.

**Figure 10:**
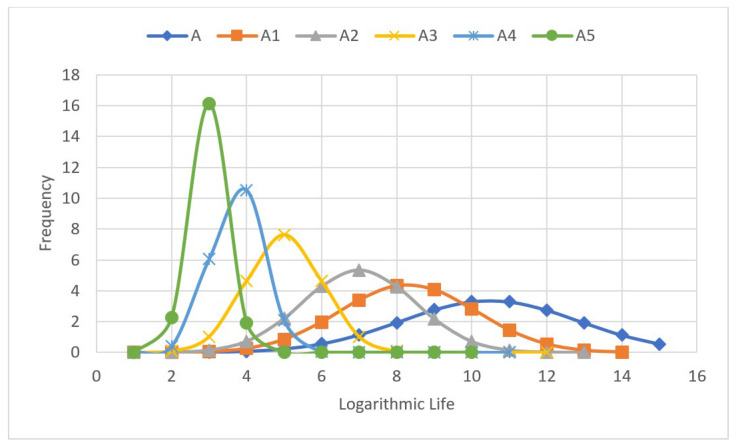
Normal distribution plot of Group A fatigue life values before and after processing to remove abnormal data.

Figure 10 shows the log-normal distribution plots for the Group A dataset before processing and the five Group A datasets after processing. Clearly, the standard deviation of the data changed significantly with ongoing processing to remove abnormal data. Indeed, the peak value of each curve increased, and the dispersion of the data decreased with ongoing processing; thus, abnormal data removal reduced the variability in the fatigue life data.

### 3.3. Hypothesis Testing

To assess how shot peening affects specimen fatigue performance, hypothesis testing was performed on the fatigue life data from three groups.

Under the same test conditions, insignificant differences in the test results are typically caused by random errors [17]. However, differences will arise in the test results if the specimen treatment method is changed from one group to another [18]; these differences include random errors as well as those induced by the change in treatment method [19]. Thus, the influence of the treatment method on fatigue performance can be determined based on the significance of these differences.

When undertaking such grouped comparative tests, researchers typically focus on the differences between average data values and occasionally on the differences in their variances (i.e., the degree of data dispersion). Hypothesis-testing methods are used to distinguish between these differences, with the *t*-test used to determine the difference between the averages of two populations and the *F*-test used to determine the difference between the variances of two populations [20]. These two methods were accordingly employed to evaluate the fatigue lives of the specimens in each treatment group [21].

The homogeneity of variances was evaluated because different methods can be used to estimate the difference between the means of two samples depending on whether or not the variances are homogeneous. Therefore, a pairwise *F*-test for the homogeneity of variances was conducted on the data for the three specimen groups using a significance level of *p* < 0.01. Table 4 presents the results of the *F*-tests. As there were significant differences between the variances of the fatigue life data for the unpeened and shot-peened specimen groups, we concluded that the variances of these data were unequal. However, there were no significant differences between the variances of the fatigue life data for the two shot-peened specimen groups, indicating that the variances of these data were equal.

The specific procedure for the *t*-test assumes a null hypothesis $H_{0}$ that the population means for the two groups of sample data are equal. This assumption allows for the calculation of test statistic *t* as follows:

(1) $$t=\frac{\bar{x_{1}}-\bar{x_{2}}}{\sqrt{\frac{\left(n_{1}-1\right)S_{1}^{2}+\left(n_{2}-1\right)S_{2}^{2}}{n_{1}+n_{2}-2}(\frac{1}{n_{1}}+\frac{1}{n_{2}})}}$$

Next, the overall degrees of freedom $\vartheta =n_{1}+n_{2}-2$ are determined, where $n_{1}$ and $n_{2}$ denote the sample sizes of each group. Subsequently, the significance level α is selected and the value of $t_{\alpha }(\vartheta )$ is obtained from the *t*-distribution table. If ${t<t}_{\alpha }(\vartheta )$, then the null hypothesis holds, and no significant difference is considered to exist between the means of the groups, indicating that both sets are the same.

A two-sample *t*-test with unequal variances was used to compare the mean fatigue lives of the unpeened, low-velocity shot-peened, and high-velocity shot-peened specimen groups, whereas a two-sample *t*-test with equal variances was used to compare the mean fatigue lives of the two shot-peened specimen groups. The results are summarized in Table 5, which indicates that there were significant differences between the means of the three groups, confirming that the shot peening process had a significant impact on the fatigue lives of the test specimens, as did the intensity of shot peening.

### 3.4. Comparison of Fatigue Reliability

The fatigue life data for military aircraft structures are typically assumed to follow a log-normal distribution [22]. Therefore, this study used the design specifications for military aircraft to calculate the basic fatigue reliability values at a reliability level of 99.9% and a confidence level of 90%, thereby comparing the fatigue performances of the three specimen groups [23]. The basic fatigue reliability ($N_{99.9/90}$) can be obtained by dividing the characteristic life $N_{50}$ of the structural component, which is derived from test or service data, by the reliability coefficient $S_{R}$, confidence coefficient $S_{C}$, and specimen coefficient $S_{T}$ [24] as follows:

(2) $$N_{99.9/90}=\frac{N_{50}}{S_{R}S_{C}S_{T}}$$

where $S_{R}=10^{\sigma_{0}\mu_{R}}$, $S_{C}=10^{\mu_{1-\alpha }\sigma_{0}/\sqrt{n}}$, and $S_{T}=1.3$, in which $\sigma_{0}$ is the standard deviation of the log-normal distribution, $\mu_{R}$ is the quantile of the log-normal distribution corresponding to the reliability R, and α is the significance level of 0.01.

The $N_{99.9/90}$ values for three specimen groups are summarized in Table 6, which clearly shows that the shot peening process significantly enhanced the specimen fatigue reliability and that low-intensity shot peening did so more effectively than high-intensity shot peening.

## 4. Conclusions

This study conducted fatigue tests on unpeened, low-intensity shot-peened, and high-intensity shot-peened 2060-T8E30 aluminum–lithium alloy specimens, then enhanced the reliability of the fatigue life results using statistical methods. The following conclusions were drawn from the analysis results:

(1) The fatigue life data were analyzed using a normality test. If such data do not satisfy the normality requirements, relevant processing is required to ensure that they conform to the normality requirements. The collected data were confirmed to follow a log-normal distribution.
(2) The outliers in the fatigue life data were removed using statistical methods to reduce the dispersion of the data, ensuring that it accurately reflected the fatigue lives of the specimen population.
(3) Overall, the scatter in the fatigue life data for the shot-peened specimens was greater than that for the unpeened specimens, and there were no significant differences between those for the low- and high-intensity shot-peened specimens.
(4) Statistical analyses indicated that shot peening significantly enhanced the fatigue life of the 2060-T8E30 alloy and that a longer fatigue life and higher fatigue reliability were achieved by low-intensity shot peening than by high-intensity shot peening.
(5) The results of this study suggest that the reliability of fatigue test results can be enhanced by improving the consistency of fatigue specimen processing.

## Figures and Tables

**Table 1:** Specimen parameters.

Specimen Type	Shot Type	Shot Peening Parameters	Specimen Thickness	Number of Specimens	Specimen Number
Unpeened	None	None	2.2	15	A-1–A-15
Low-intensity shot peening	Cast steel shot S230	0.20 ± 0.038 mmN 100% coverage rate	2.2	15	B-1–B-15
High-intensity shot peening	Cast steel shot S230	0.36 ± 0.038 mmN 100% coverage rate	2.2	15	C-1–C-15

**Table 2:** Fatigue life data obtained from specimen tests.

Specimen	Fatigue Life (Cycles)	Specimen	Fatigue Life (Cycles)	Specimen	Fatigue Life (Cycles)
A-1	53,448	B-1	527,107	C-1	506,645
A-2	122,414	B-2	390,764	C-2	115,496
A-3	57,546	B-3	751,497	C-3	245,784
A-4	75,144	B-4	466,083	C-4	140,810
A-5	94,246	B-5	483,501	C-5	151,212
A-6	57,969	B-6	660,933	C-6	107,832
A-7	53,764	B-7	674,324	C-7	181,393
A-8	54,907	B-8	227,399	C--8	146,518
A-9	56,691	B-9	738,583	C-9	175,174
A-10	54,553	B-10	199,481	C-10	114,756
A-11	62,018	B-11	607,939	C-11	132,780
A-12	45,427	B-12	380,145	C-12	146,109
A-13	89,023	B-13	217,562	C-13	611,898
A-14	50,154	B-14	598,236	C-14	260,341
A-15	56,260	B-15	678,816	C-15	244,730

**Table 3:** Abnormal data processing results for each group.

Specimen Group	i	Logarithmic Mean	Logarithmic Standard Deviation	Maximum Deviation Ratio	Processing Result	Post-Processed Group	Proportion of Outlier Data
A	2	4.80	0.12	2.13	Discard	A1	33%
A1	5	4.78	0.09	2.10	Discard	A2	
A2	13	4.76	0.07	2.07	Discard	A3	
A3	4	4.75	0.05	2.03	Discard	A4	
A4	12	4.74	0.04	2.00	Discard	A5	
B	10	5.67	0.20	2.13	Keep	B	0
C	13	5.28	0.23	2.13	Discard	C1	6.7%
C1	1	5.24	0.18	2.10	Discard	C2	

**Table 4:** Results of the homogeneity test of variance for the three fatigue life groups.

Comparison	One-Tailed *p*-Value	One-Tailed Critical Value of *t*	Judgment	Conclusion
A5 and B	1.2 × 10^−6^	0.17	*p* < 0.01	Falls into the rejection region, indicating a significant difference in variances
A5 and C2	1.32 × 10^−5^	0.19564	*p* < 0.01	Falls into the rejection region, indicating a significant difference in variances
B and C2	0.0655	4.341624	*p* > 0.01	Does not fall into the rejection region, indicating no significant difference in variances

**Table 5:** Results of the *t*-test for the three fatigue life groups.

Comparison	Test Method	Two-Tailed *p*-Value	Two-Tailed Critical Value of *t*	Judgment	Conclusion
A5 and B	Two-sample *t*-test with unequal variances	1.51 × 10^−11^	2.94	*p* < 0.01	There is a significant difference between the means.
A5 and C2	Two-sample *t*-test with unequal variances	1.37 × 10^−8^	3.01	*p* < 0.01	There is a significant difference between the means.
B and C2	Two-sample *t*-test with equal variances	1.02 × 10^−7^	2.77	*p* < 0.01	There is a significant difference between the means.

**Table 6:** Basic fatigue reliability $N_{99.9/90}$ statistics for the three specimen groups.

Statistic	Unpeened Group A5	Low-Intensity Shot-Peened Group B	High-Intensity Shot-Peened Group C2
$N_{50}$	55,731	506,825	166,380
$S_{R}$	1.31	3.50	1.80
$S_{C}$	1.01	1.10	1.07
$S_{T}$	1.00	1.00	1.00
$N_{99.9/90}$	41,911	131,617	86,610

## Data Availability

The original contributions presented in the study are included in the article, further inquiries can be directed to the corresponding author.
